# Use of 4-factor prothrombin complex concentrate in the treatment of a gastrointestinal hemorrhage complicated by dabigatran

**DOI:** 10.1186/s12245-015-0059-2

**Published:** 2015-04-15

**Authors:** Terrance R McGovern, Justin J McNamee, Christopher Malabanan, Mohamed A Fouad, Nilesh Patel

**Affiliations:** Department of Emergency Medicine, St. Joseph’s Regional Medical Center, 703 Main Street, Paterson, NJ 07503 USA; Department of Pharmacy, St. Joseph’s Regional Medical Center, 703 Main Street, Paterson, NJ 07503 USA

**Keywords:** Emergency medicine, Target-specific oral anticoagulants, Dabigatran, Prothrombin complex concentrate, Reversal

## Abstract

Target-specific oral anticoagulants (TSOACs) provide patients and healthcare providers with an alternative to vitamin K antagonists (VKA). The TSOACs are of similar or superior efficacy to warfarin, but unlike VKAs, there are no approved ‘antidotes’ for rapid reversal of life-threatening bleeding on therapy. We report here the case of an 83-year-old gentleman, who presented to the emergency department with severe gastrointestinal hemorrhage and coagulopathy (hemoglobin: 5.3 g/dL and INR: 2.2) while on the direct thrombin inhibitor dabigatran. His coagulopathy reversed rapidly after administration of 4-factor prothrombin complex concentrate (4 F-PCC), and after initial administration of 2 units of packed red blood cells, no further product transfusions were required. He was discharged 4 days later without further complications.

## Background

The U.S Food and Drug Administration (FDA) approved dabigatran etexilate for prevention of stroke in patients with nonvalvular atrial fibrillation, as well as for treatment and prevention of recurrent venous thromboembolism (VTE) in previously treated patients [1]. Dabigatran, 150 mg twice a day, has shown superiority over warfarin with respect to its ability to prevent stroke with a lower rate of life-threatening bleeding complications; however, dabigatran has an increased risk of gastrointestinal bleeding [2]. Similar to all target-specific oral anticoagulants (TSOACs) without a reversal agent, unintentional bleeding remains a significant risk of treatment. There has been some promise demonstrated in the use of 4-factor prothrombin complex concentrate (4 F-PCC) in dabigatran-associated hemorrhage in a recent case series [3]. Here, we present a patient on dabigatran who presented to the emergency department (ED) with severe gastrointestinal hemorrhage, which was treated with 4-factor prothrombin complex concentrate.

## Case presentation

An 83-year-old male with previous medical history of hypertension, hyperlipidemia, coronary artery disease, paroxysmal atrial fibrillation, transient ischemic attacks, and a left atrial thrombus presented to the emergency department with complaints of aphasia and bilateral lower extremity weakness that began 1 h prior to arrival. While *en route* to the hospital, the patient’s aphasia and lower extremity weakness had resolved, and on arrival to the ED, he had no acute complaints. On further questioning, the patient states that he had noticed malodorous bowel movements and hematochezia for the past 5 days. At the time of presentation, the patient’s vital signs were blood pressure: 128/51, pulse: 77, respiratory rate: 18, pulse oximetry: 99%, and temperature: 100.6 °F. The patient’s physical exam was significant for pallor, +2/4 systolic murmur, bibasilar rales on lung auscultation, and guaic-positive maroon stool on rectal exam. The patient’s home medication regimen was digoxin 0.125 mg daily, dronedarone 400 mg twice a day (BID), atorvastatin 40 mg daily, enalapril 20 mg daily, metoprolol succinate 40 mg daily, pantoprazole 40 mg daily, and dabigatran 150 mg BID. He stated that he was adherent to his medications, including dabigatran, and had taken the last dose 4 h prior to arrival. Table 1 illustrates the patient’s routine laboratory analysis at initial presentation.

**Table 1:** **Patient’s laboratory data**

**Lab**	**Day 0 (ED)**	**Day 1**	**Day 4 (discharge)**
WBC	11.2 K/mm^3^	-	-
Hgb	5.3 g/dL	7.6 g/dL	7.8 g/dL
Hct	15.7 g/dL	22.9 g/dL	23.4 g/dL
Platelet	142 K/mm^3^	-	-
Na	162 meq/L	-	-
K	5.4 meq/L	-	-
Cl	127 meq/L	-	-
CO2	23 meq/L	-	-
BUN	39 mg/dL	-	-
Cr	1.45 mg/dL	-	-
Glucose	171 mg/dL	-	-
CrCl	35 to 40 mL/min	-	-
PT	24.0 s	18.1 s	16.7 s
INR	2.2	1.5	1.3
aPTT	57.2 s	38.6 s	35.4 s

Patient’s initial laboratory values in the emergency department and subsequent hemoglobin/hematocrit and coagulation profile throughout the patient’s inpatient hospitalization. CrCl, creatinine clearance; PT, prothrombin time; INR, international normalized ratio; aPTT, activated partial thromboplastin time; Hgb, hemoglobin; Hct, hematocrit; BUN, blood urea nitrogen.

Based on the patient’s history, severe anemia and suspected gastrointestinal hemorrhage, he was transfused 2 units of packed red blood cells and started on a proton pump inhibitor; arguably, the most pressing issue was reversing the patient’s coagulopathy. To replenish the patient’s coagulation factors, he was given a dose of 25 units/kg (72 kg; 1,800 units) of 4 F-PCC based on institution protocol. Once stabilized in the ED, the patient was admitted to the medical intensive care unit for further evaluation and treatment. The patient’s repeat coagulation profile the next day, after the administration of the 4 F-PCC, showed an improvement in his coagulation factors to prothrombin time (PT): 18.1 s, international normalized ratio (INR): 1.5, and activated partial thromboplastin time (aPTT): 38.6 s and stabilization of his hemoglobin at 7.6 g/dL and hematocrit to 22.9 g/dL after the 2 units of packed red blood cells in the ED (Table 1). Upper endoscopy revealed no active sites of bleeding, and he did not require any further blood transfusions during his inpatient hospitalization. He was subsequently discharged 4 days later after discontinuing his dabigatran with a normalized coagulation profile and stabilized anemia as demonstrated in Table 1.

## Discussion

Dabigatran acts as a potent anticoagulant by preventing thrombin’s conversion of fibrinogen to fibrin and inhibits the ability of thrombin to activate factors V, VIII, and XI. In patients with normal renal function (creatinine clearance (CrCl) > 80 mL/min), dabigatran has a half-life of 13.8 h; however, when the CrCl < 30 mL/min, the half-life extends to 27.5 h [4]. Dabigatran is primarily (80%) renally excreted and reaches its peak concentration in about 2 h [4,5].

In patients taking warfarin, evaluating the INR provides a quantitative means of assessing the extent of anticoagulation for the patient. All coagulation assays may be affected by dabigatran; however, the most common assays lack sensitivity in determining the extent of anticoagulation. The PT and INR are both insensitive in determining the extent of dabigatran activity, but there can be small elevations in the patient’s INR in supratherapeutic ranges of dabigatran [6]. Thrombin clotting time (TT) and ecarin clotting time (ECT) are the most accurate and proportionally increase up to a value of 400 ng/mL of dabigatran but are not routinely available in the emergency department [6]. The most readily available coagulation assay in the ED for monitoring the anticoagulant effect of dabigatran is the aPTT. The dose–response curve is curvilinear when looking at the drug concentration and corresponding increase in aPTT [6]. While aPTT cannot be relied upon to provide an exact measurement of dabigatran in the blood, it can be qualitatively used to determine excessive anticoagulation.

Dabigatran is a viable alternative to warfarin for prevention of stroke and systemic thromboembolism in patients with nonvalvular atrial fibrillation with Class IB evidence from the current 2014 AHA/ASA/HRS recommendations [7]. Additionally, the use of dabigatran was associated with lower risk of life-threatening and intracranial bleeding when compared to warfarin; however, the randomized evaluation of long-term anticoagulation therapy (RE-LY) trial and a recent meta-analysis of randomized controlled trials on the bleeding risk of dabigatran demonstrated an increased risk for gastrointestinal bleeding (RR: 1.51, 95% CI 1.23 to 1.84) [2,8].

4-factor prothrombin complex concentrate is indicated for urgent reversal of vitamin K antagonists, such as warfarin, by providing the vitamin-K-dependent factors II, VII, IX, and X as well as protein C and S. Theoretically, the high concentration of these coagulation factors should assist in thrombin activation and subsequent clot formation. Experimental results have been variable when analyzing nonactivated 4 F-PCC’s efficacy in treating dabigatran-related bleeds. In animal models, there was an improvement in bleeding time, but there was no effect on the coagulation assays [9,10]. In 2015, Hoffman et al. used a cell-based model to demonstrate improvement of multiple parameters of thrombin generation after treatment with prothrombin complex concentrate in the presence of therapeutic levels of dabigatran [11]. In a murine model of dabigatran-induced coagulopathic mice, prothrombin complex concentrate did not significantly reduce blood loss after a tail transection [12]. In a separate murine model, prothrombin complex concentrate was able to prevent further intracerebral hematoma growth in mice treated with dabigatran [13]. A 12-patient, randomized, double-blind, placebo-controlled crossover study evaluated the effects of 4 F-PCC on both dabigatran and rivaroxaban reversal. The 12 healthy patients randomly received dabigatran or rivaroxaban for 2.5 days and then received 4 F-PCC (50 units/kg) or a placebo. After an 11-day washout period, the subjects received the other anticoagulant for 2.5 days and again received either the 4 F-PCC (50 units/kg) or a placebo. After comparing the aPTT, endogenous thrombin potential (ETP), lag time, TT, and ECT in all the subjects, the administration of the 4 F-PCC had no influence on these coagulation assays for the patients receiving dabigatran [14]. With the lack of consensus and variability of results between animal models, case reports, and small human trials, a group of experts composed of members from the American Society of Health-System Pharmacists (ASHP) met to propose management considerations for patients bleeding while on TSOAC [15]. After reviewing the available literature, their first choice for reversal of dabigatran was activated PCC (aPCC); however, their second choice was nonactivated 4 F-PCC, both of which are recommended with the acknowledgement that their recommendation is based on limited available clinical data [15]. The Thrombosis and Hemostasis Summit of North America (THSNA) in 2012 also recognized the paucity of data in human trials and could not come to a consensus on prothrombin complex concentrate’s role in reversal of dabigatran; however, some authors did feel that it would be reasonable in certain circumstances [16].

Despite conflicting data and case reports regarding the utility of 4 F-PCC in the treatment of bleeding while on dabigatran, the case presented here demonstrated a normalization of the patient’s coagulation profile, stabilization of his hemoglobin, and a positive clinical outcome of the patient being discharged home without incurring any further morbidity from his condition. The extent of the patient’s hemorrhage had not reached the point of causing objective signs of hemodynamic instability, possibly due to his beta-blocker home medication. Nonetheless, having a hemoglobin of 5.3 g/dL in a patient with significant cardiovascular pathology was deemed reason enough to proceed with attempting to rapidly reverse the coagulopathic effect of dabigatran with 4 F-PCC*.* The patient may have been inadvertently supratherapeutic due to his acute renal insufficiency (CrCl: 35 to 40 mL/min). Dabigatran is renally excreted, and in patients with renal impairment (CrCl < 30 mL/min), the half-life can be prolonged up to 27.5 h [4]. In certain circumstances where there is significant renal dysfunction with a life-threatening bleed, dabigatran can be dialyzed because of its low plasma protein binding. It has been shown that up to 62% of a single 50-mg dose of dabigatran can be removed by dialysis in 2 h in end-stage-renal-disease patients on hemodialysis; this percentage increased to 68% at 4 h [4]. Due to relative inexperience with emergent extracorporeal therapy for dabigatran removal in life-threatening bleeds, prior case reports have discovered a ‘rebound’ level of dabigatran after initial hemodialysis. Dabigatran levels rebound in the terminal elimination phase due to the large volume of distribution reportedly as high as 87% in one single-center case series causing a trend in coagulation assay levels to pre-hemodialysis levels. Based on the rebound phenomena, the latest recommendation for hemodialysis for dabigatran reversal is either prolonged intermittent hemodialysis or initial intermittent hemodialysis followed by continuous renal replacement therapy [17,18]. In the case of life-threatening hemorrhage but without significant renal impairment, such as our patient, hemodialysis is less desired and other reversal agents (i.e., 4 F-PCC) need to be explored and considered.

Another consideration is the drug interaction that exists between dabigatran and dronedarone, which were both taken by the patient prior to his hospitalization. Dronedarone is a P-glycoprotein inhibitor while dabigatran is a p-glycoprotein substrate. Co-administration can increase dabigatran exposure up to 99%, leading to the manufacturer’s recommendation to reduce the dabigatran dose to 75 mg BID in moderate renal impairment (CrCl 30 to 50 mL/min) [1,19]. The patient receiving full dose dabigatran 150 mg BID while also sustaining acute renal injury also contributed to excessive anticoagulation.

There remains no validated reversal agent to dabigatran-related coagulopathy, but there may be soon. Currently undergoing phase III clinical trials, idarucizumab is a monoclonal antibody that targets dabigatran and may be a potential solution. Two *ex vivo* studies comparing the use of PCCs and idarucizumab’s ability to reverse dabigatran-related bleeding demonstrated that idarucizumab corrected PT and aPTT and reduced the plasma concentration of dabigatran to zero [20,21]. Additionally, the first-in-human study also yielded positive results and marked idarucizumab safe and tolerable in healthy males [22].

## Conclusions

TSOACs provide an alternative choice to warfarin for oral anticoagulation and will likely continue to be utilized by physicians and their patients. Until there is a commercially available ‘antidote’ to directly counteract the TSOACs quickly and effectively, the risk of significant or catastrophic hemorrhage remains. Here, we presented a case where 4-factor prothrombin complex concentrate was used to expeditiously reverse dabigatran in a patient with severe anemia due to gastrointestinal hemorrhage. While more robust studies are needed to determine the best method of reversal for TSOACs, the use of 4-factor prothrombin complex concentrate should be considered in situations where it is necessary to counter the hemorrhagic complications of dabigatran.

## Consent

Written informed consent was obtained from the patient for publication of this case report and any accompanying images. A copy of the written consent is available for review by the Editor-in-Chief of this journal.

## Abbreviations

4 F-PCC: 4-factor prothrombin complex concentrate
aPCC: activated prothrombin complex concentrate
aPTT: activated partial thromboplastin time
ASHP: American Society of Health-System Pharmacists
BID: twice daily
CrCl: creatinine clearance
ECT: ecarin clotting time
ED: emergency department
ETP: endogenous thrombin potential
FDA: Food and Drug Administration
INR: international normalized ratio
PT: prothrombin time
TSOAC: target-specific oral anticoagulant
TT: thrombin clotting time
VKA: vitamin K antagonist
VTE: venous thromboembolism
